# Validation pipeline for machine learning algorithm assessment for multiple vendors

**DOI:** 10.1371/journal.pone.0267213

**Published:** 2022-04-29

**Authors:** Bernardo C. Bizzo, Shadi Ebrahimian, Mark E. Walters, Mark H. Michalski, Katherine P. Andriole, Keith J. Dreyer, Mannudeep K. Kalra, Tarik Alkasab, Subba R. Digumarthy

**Affiliations:** 1 MGH & BWH Center for Clinical Data Science, Mass General Brigham, Boston, Massachusetts, United States of America; 2 Department of Radiology, Massachusetts General Hospital, Harvard Medical School, Boston, Massachusetts, United States of America; 3 Department of Radiology, Brigham and Women’s Hospital, Harvard Medical School, Boston, Massachusetts, United States of America; University of Groningen, University Medical Center Groningen, NETHERLANDS

## Abstract

A standardized objective evaluation method is needed to compare machine learning (ML) algorithms as these tools become available for clinical use. Therefore, we designed, built, and tested an evaluation pipeline with the goal of normalizing performance measurement of independently developed algorithms, using a common test dataset of our clinical imaging. Three vendor applications for detecting solid, part-solid, and groundglass lung nodules in chest CT examinations were assessed in this retrospective study using our data-preprocessing and algorithm assessment chain. The pipeline included tools for image cohort creation and de-identification; report and image annotation for ground-truth labeling; server partitioning to receive vendor “black box” algorithms and to enable model testing on our internal clinical data (100 chest CTs with 243 nodules) from within our security firewall; model validation and result visualization; and performance assessment calculating algorithm recall, precision, and receiver operating characteristic curves (ROC). Algorithm true positives, false positives, false negatives, recall, and precision for detecting lung nodules were as follows: Vendor-1 (194, 23, 49, 0.80, 0.89); Vendor-2 (182, 270, 61, 0.75, 0.40); Vendor-3 (75, 120, 168, 0.32, 0.39). The AUCs for detection of solid (0.61–0.74), groundglass (0.66–0.86) and part-solid (0.52–0.86) nodules varied between the three vendors. Our ML model validation pipeline enabled testing of multi-vendor algorithms within the institutional firewall. Wide variations in algorithm performance for detection as well as classification of lung nodules justifies the premise for a standardized objective ML algorithm evaluation process.

## Introduction

Interest in machine learning (ML) algorithms for use in radiology is growing rapidly. As algorithms become available for clinical use, an objective method for evaluating these tools is required. ML challenges are one mechanism for assessing algorithm performance, and such competitions involving medical images are increasing. Recent examples include the RSNA Pediatric Bone Age Challenge [1], Data Science Bowl 2017 [2], Ischemic stroke lesion segmentation (ISLES) Challenge [3], Multimodal Brain Tumor Image Segmentation Benchmark (BRATS) [4], and Lung Nodule Analysis 2016 (LUNA16) challenge [5], among others. Competitions are open and generally provide a public annotated data set for the training and evaluation of participant algorithms.

Such open challenges are an objective way for the research community to develop, test, compare, and advance state-of-the-art data science and ML techniques. However, a large gap exists between algorithm performance evaluated on a highly-curated research dataset and a clinically usable product that provides valuable advice in real-world use. Success of ML algorithms on research datasets from specific site (s), timepoints, imaging equipment and acquisition parameters, as well as disease type, spectrum, severity, and presentation, is not a guarantee of its generalizability, explainability, robustness, or performance in clinical environment. To address these issues, it is important to develop and incorporate objective processes to validate ML algorithms on representative local clinical data.

There is an urgent need for an objective, standardized evaluation process to compare algorithms on an even clinical plane. For this purpose, we designed, built, and tested an evaluation pipeline for the use case of lung nodule detection on chest CT studies.

Lung cancer is currently the leading cause of cancer death in the US [6]. There is a substantial economic burden associated with lung cancer care, with an overall cost estimated in 2016 at over $13 billion [7]. Further, a 2015 Centers for Medicare and Medicaid Services mandate requires a standardized identification, classification, and reporting system to report lung cancer screening imaging studies for reimbursement [8]. In this context, CT chest screening examinations are increasing in number, and many companies have developed nodule detection software tools in hopes of early lung nodule detection for improved patient outcomes.

Along with the socio-economic reasons, the high vendor interest in systems for automatic detection of lung nodules makes assessing such tools a valuable use case. However, the heterogeneity of existing systems’ capabilities, the substantial variability among radiologists on what constitutes a lung nodule [9], and the coexistence of other pathologic pulmonary imaging findings highlight the inherent challenges to building an evaluation system. Therefore, our goal was to normalize clinical assessment of different lung nodule detection machine learning algorithms using a standard evaluation pipeline generalizable to other modalities and clinical evaluations.

## Materials and methods

This retrospective study was approved by Mass General Brigham Institutional Review Board (Approval number: 2016P000312). The need for consent was waived by the ethics committee. It was performed at an urban tertiary care academic medical center. Our data-preprocessing and algorithm assessment chain included the following components 1. an image cohort creation and de-identification tool 2. report and image annotation tools for ground-truth labeling 3. server partitioning for vendor “black box” algorithms, and model testing on our internal clinical data within our security firewall 4. model validation and result visualization tool and 5. assessment of algorithm recall and precision. This section provides a brief description of each step required for building, implementing, and testing the evaluation pipeline.

### Data collection

**Fig 1:**
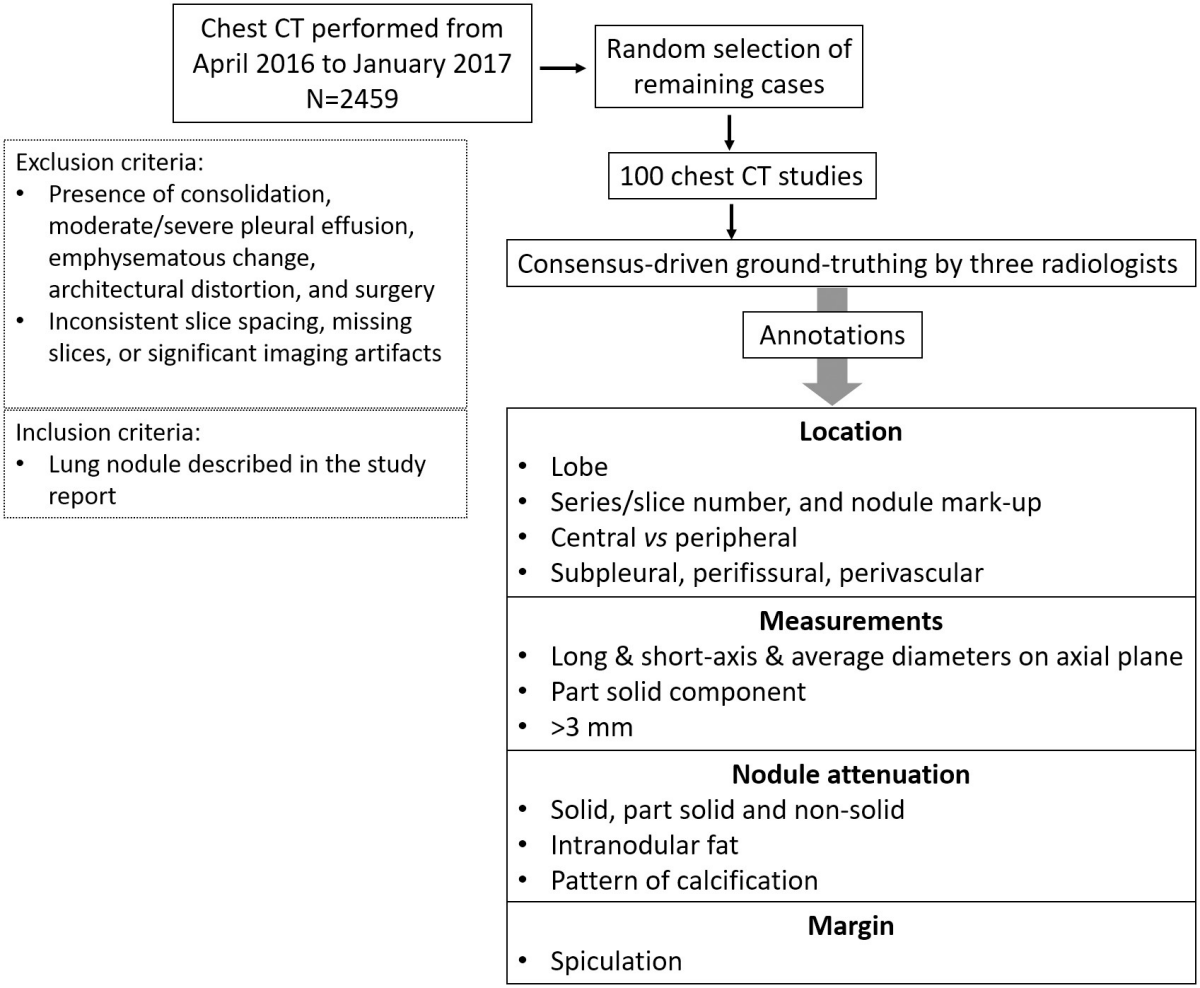
Flowchart of patient population in the study. It is summarizing the ground-truthing of pulmonary nodules by three radiologists.

Cohort selection was made on picture archiving and communication system (PACS) (AGFA Technical Imaging Systems, Ridgefield Park, NJ). Two thousand four hundred fifty-nine (2459 CTs) consecutive chest CT studies and reports were reviewed to include 100 CT studies done for lung nodule surveillance or detection (Fig 1). The CTs were performed on ten different multi-slice CT scanner models with 16 to 256 channels from three different manufacturers during the time frame from April 2016 to January 2017. The selected 100 CTs in 100 different patients (46 male, 54 female: age 19 to 92 years old) were acquired with slice thickness ≤ 3 mm (18 studies with 1 mm, 23 studies with 1.25 mm, 54 studies with 2.5 mm, and 5 studies with 3 mm), with regular or low dose acquisition, with (46 studies) or without (54 studies) intravenous contrast, and different reconstruction methods (e.g. standard and over-enhancing kernels such as detail, I30f, I31f, B30f and B31f).

### Report and image annotation

To select the final cohort of 100 chest CT studies with lung nodules from the larger cohort of 2459 chest CT studies retrieved from our PACS archive, two infrastructure software applications were built in-house: a report annotation tool and an image annotation-visualization tool to facilitate the process in a semi-automated fashion.

The web-based interactive report annotation tool consisted of a graphical user interface that enabled report loading and user-configurable search terms. The tool allows for the assignment of multiple study-level labels per report and keyword or phrase search. The presence of specific wording in the study report can be used as an initial filter to select studies likely to present determinate imaging findings. Specifically, using the tool, a radiologist views a report, highlights a phrase or keyword of interest, and associates it with a given label. Any report containing this phrase or keyword, and not previously matched to another phrase, is marked with a “soft label.” This process allows the radiologist to cull many reports and group them efficiently. Next, manual confirmation of the initial soft labels is performed on a study-by-study basis, and exclusion criteria are applied. This preliminary grouping of reports facilitates and expedites further processing.

Most current ML algorithms (2/3 assessed AI algorithms) are limited in detecting a single imaging finding—so-called “narrow AI” (artificial intelligence). Therefore, studies containing other non-nodule-relevant imaging findings were not included in the final cohort to properly assess the nodule detection capabilities of the evaluated models. Instead, study-level labels were created in the report annotation tool, and keyword searches of related terms were used to identify and consequently exclude reports containing variations of the following excluded criteria: consolidation, moderate/severe pleural effusion, emphysematous change, architectural distortion, and surgery. After removing study reports containing these findings, the remaining reports were assessed for a first pass filtering of studies with mention of lung nodules in the reports (keyword searches for solid, part-solid, and ground glass nodules).

This method was applied to all 2459 study reports, and 100 studies containing nodules of at least one composition and without keywords or phrases related to the exclusion criteria were randomly selected. A radiologist reviewed all images to confirm the presence of lung nodule(s) without significant possible confounding factors (excluding cases that had findings described in the exclusion criteria that might not have been described in the radiology report text but were evident on image review) and assessed for quality assurance. Studies with inconsistent slice spacing, missing slices, or significant artifacts from motion or metal were excluded. All images were stripped of identifying patient information and re-coded with study-specific numbers, with the mapping blind to all but the study project manager.

Usable studies were loaded into our image annotation tool. Three radiologists annotated each CT using the web-based application developed using open-source and commercial tools. The Cornerstone Javascript library was used to render images and annotation markup, Microsoft. NET 4.5 application programming interface for endpoint development, Microsoft’s Internet Information Services for hosting, and Microsoft’s SQL Server Express for application persistence. The resulting image display tool included window-width/level manipulation, zoom/pan, study/series navigation, and measurements. The measuring tools allowed bi-directional measurement (for measuring longest and shortest dimensions of all nodules) and two sets of measurements for part-solid nodules (independent of the whole nodule and separately for the solid component) that are automatically recorded and linked to identified nodules. Associated with each annotation are configurable labels defined by an Extensible Markup Language (XML) schema based on the computer-assisted reporting and decision support (CAR/DS) schema [10] that allows users to assign more nodule attributes details such as composition, location, and presence of spiculation. In addition, the schema was configured to capture information about series/slice numbers, nodule dimensions and coordinates.

Ground-truth was consensus-driven: only nodules accepted by the radiologist majority (at least 2 out of 3) were eligible for evaluation. Nodules with different compositions (i.e., solid, sub-solid, and ground glass), size ≥ 4 mm and ≤ 30 mm were annotated. Nodule measurements were performed using the lung window display. We excluded diffusely calcified nodules from the annotation process and communicated to the vendors that their system should not detect these nodules. Nevertheless, a total of eight diffusely calcified nodules were detected (three by vendor 1, four by vendor 2, and five by vendor 3). These detected granulomas were not included in the statistical analysis. The radiologists annotated the following data elements for each nodule in the ground truth data set: size (long- and short-axis diameters in the axial plane and the average, the latter calculated automatically), presence of spiculation (yes/no), and laterality and lobar location. The ground truth radiologists did not have access to radiology reports before or during annotating the ground truth.

### Data workflow process

**Fig 2:**
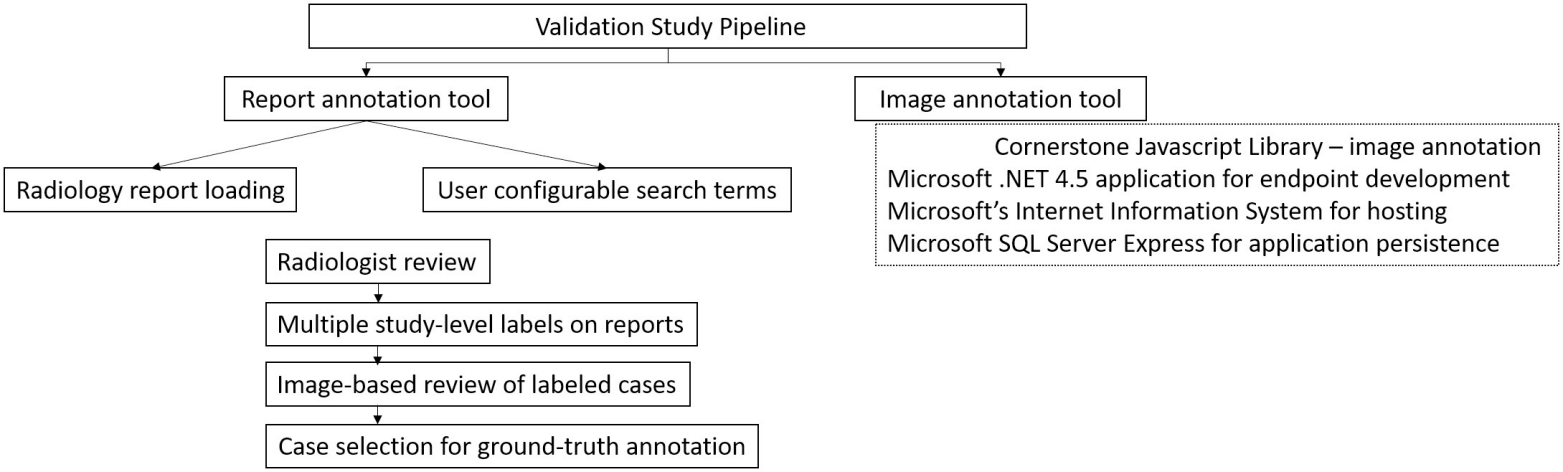
Infrastructure of validation pipeline for AI algorithms assessed in our study.

**Fig 3:**
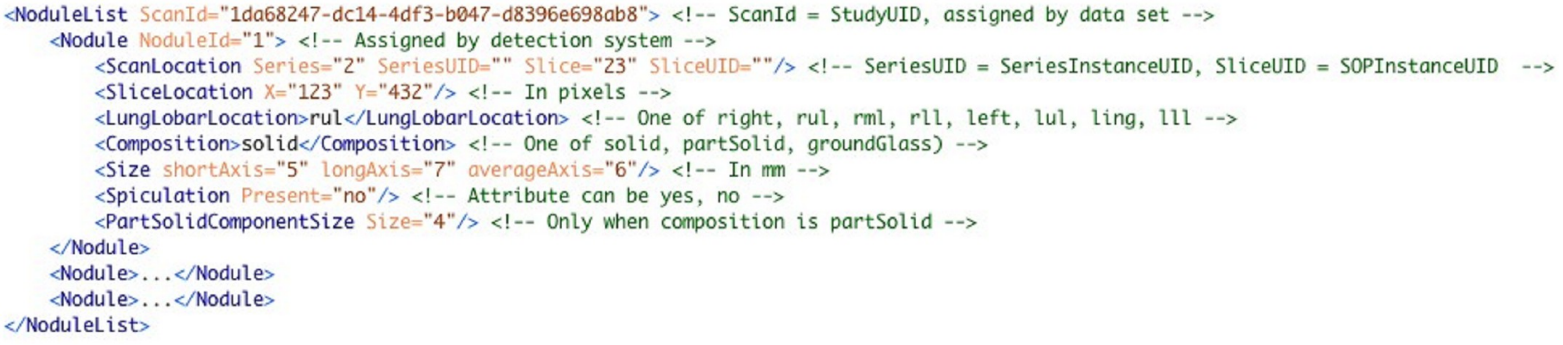
Vendor output data schema defined in Extensible Markup Language (XML).

We received each vendor algorithm wrapped in a Docker container that was loaded on individual servers. Our chest CT dataset is input to the vendor algorithms for processing. Vendors were instructed to provide their output using our defined XML data schema format (Fig 2). All studies were assessed by each vendor algorithm independently, and the output XML files were stored for subsequent loading into our image annotation visualization tool for comparison to ground-truth (Fig 3).

### Evaluation process and statistical analysis

**Fig 4:**
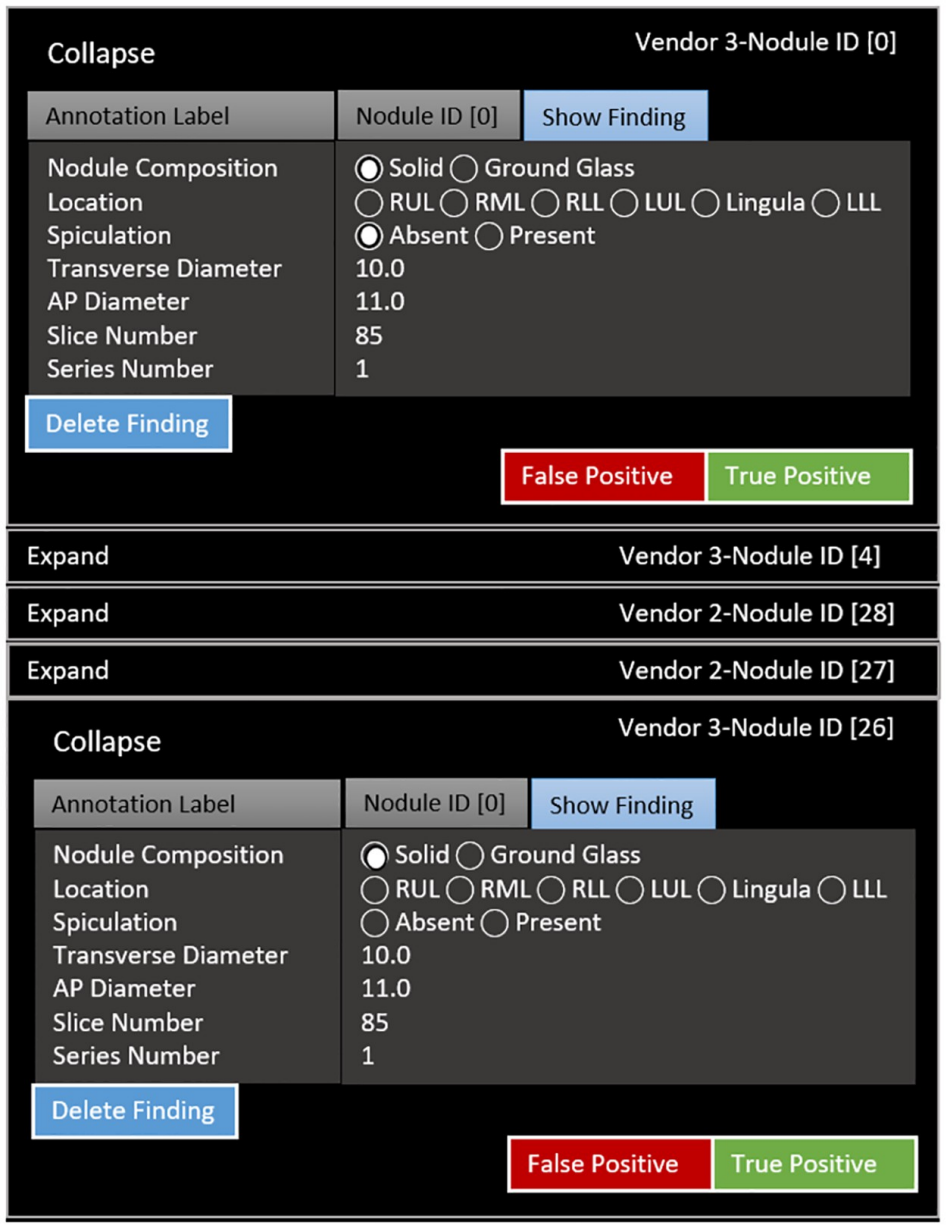
Algorithm evaluation results viewed using image annotation-visualization tool for comparison to ground-truth. Note patient protected health information (PHI) is changed for de-identification purposes.

**Fig 5:**
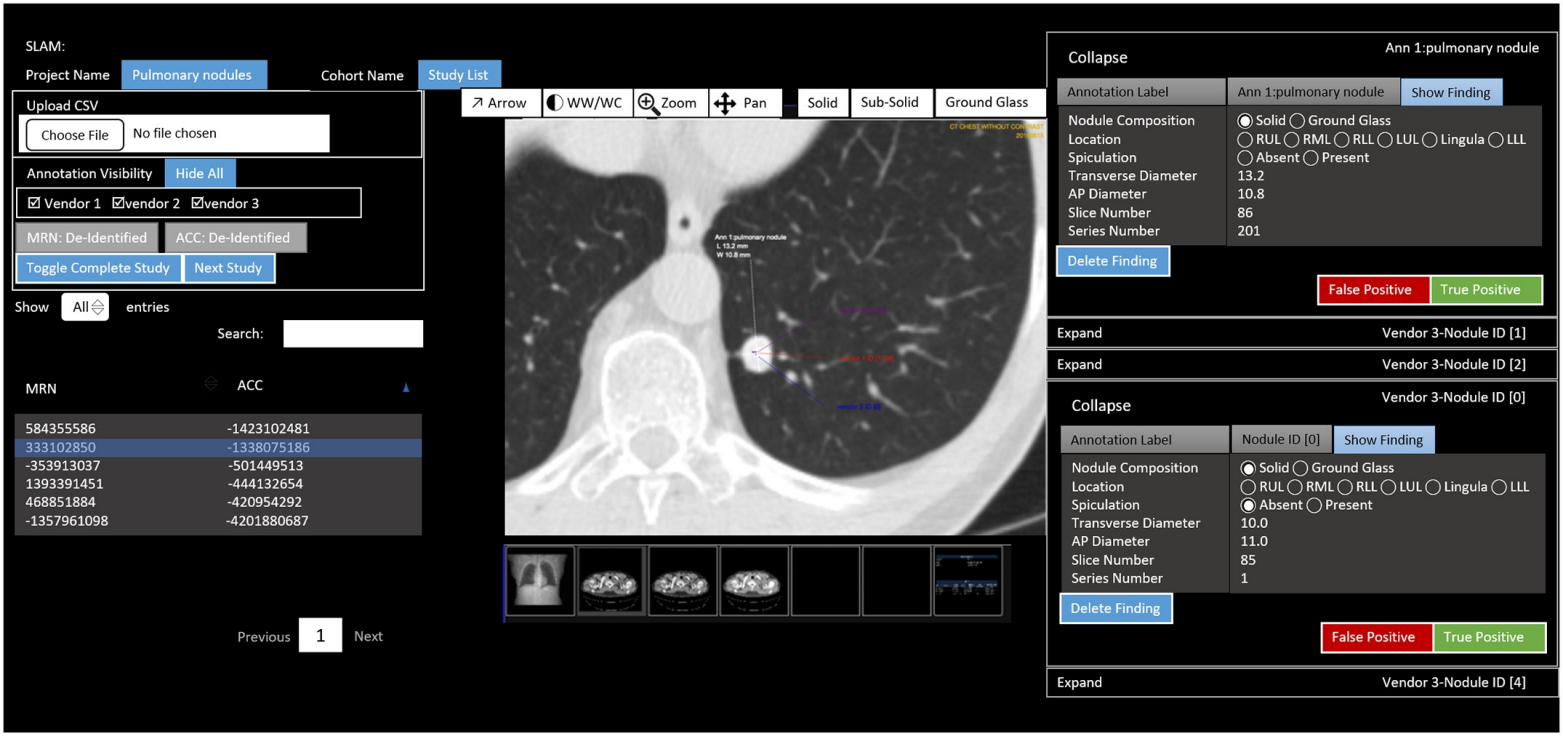
Image annotation-visualization tool showing zoom into features of vendor-detected nodules.

Manual evaluation of vendor model output versus ground-truth was performed using the image annotation visualization tool to navigate ground-truth and vendor-detected nodules (Figs 4 and 5). Each vendor-detected nodule was assessed for correlation with ground truth. A nodule was considered a true positive detection within 1 cm (calculated in all three dimensions) of ground-truth nodule locations. In addition, to be considered as true positive, the vendor predicted nodule and its annotation should overlap or intersect with the ground truth nodule, otherwise, it was considered as false positive. Otherwise, a vendor-detected nodule was considered a false positive. Each ground-truth nodule without a corresponding vendor-detected nodule was considered a false negative. A confusion matrix was tabulated using this classification scheme (Table 1).

**Table 1:** Vendor lung nodules detection performance.

	Vendor 1	Vendor 2	Vendor 3
True positives	194	182	75
False positives	23	270	120
False negatives	49	61	168
Recall	0.80	0.75	0.32
Precision	0.89	0.40	0.39

Statistical analysis was performed in Microsoft Excel and SPSS Statistical Software (Version 26, IBM Inc.) We assessed the performance of individual models with recall and precision for overall lung nodule detection. In addition, we calculated the receiver operating characteristic curves (ROC) area under the curve (AUC) with 95% confidence intervals for attenuation-based stratification of lung nodules.

## Results

A total of 243 nodules were annotated on our ground-truth dataset (127 solid, 76 ground glass, and 40 part-solid). We used medians for lung nodule size due to non-normal distribution of data. The mean average nodule size (regardless of nodule type) was 7.00 mm (median 5.65 mm; interquartile range [IQR] 4.00–22.75).

There was a significant difference in sizes of solid (median 4.77 mm, IQR 1.80 mm), part-solid (8.85 mm, 4.51 mm), and groundglass nodules (6.05 mm, 2.78 mm) (p<0.001). The detailed distribution of annotated features of the nodules is described in Table 2. Although automated nodule size calculation was present across all vendors, their models did not have the same capabilities for detecting all annotated features, such as calculating part-solid component long- and short-axis diameters and detecting lung nodule lobar location, laterality, and the presence of spiculation. Therefore, our analysis assessed only the vendor nodule detection and classification feature.

**Table 2:** Lung nodules cohort features distribution.

Variable	# of nodules (n = 243)	Frequency
**Location**		
RUL	63	25.92%
RLL	52	21.39%
RML	16	6.58%
LUL	45	18.51%
LLL	61	25.10%
Lingula	6	2.46%
**Attenuation**		
Solid	127	52.26%
Ground glass	76	31.27%
Part-solid	40	16.46%
**Spiculation**		
Yes	9	3.70%
No	234	96.29%

*RUL: Right upper lung; RLL: Right lower lung; RML: Right middle lung; LUL: Left upper lung; LLL: Left lower lung

Vendor model performance varied widely, as can be seen in Table 1. Algorithms recall and precision for detecting lung nodules were as follows: Vendor-1 (0.80, 0.89); Vendor-2 (0.75, 0.40); Vendor-3 (0.32, 0.39).

Detection of nodules of different composition was also assessed individually (Table 3). When examining vendor performance only for solid nodules (n = 127) detection, algorithm TP, FP, FN, recall, and precision were as follows: Vendor-1 (105, 22, 16, 0.82, 0.86); Vendor-2 (96, 31, 184, 0.75, 0.34); Vendor-3 (53, 74, 82, 0.41, 0.39). When ground glass only nodule detection was assessed, the same performance indicators were as follow: Vendor-1 (53, 23, 11, 0.69, 0.82); Vendor-2 (50, 26, 154, 0.65, 0.24); Vendor-3 (1, 75, 53, 0.01, 0.01). And finally, when part-solid nodule detection was assessed, results were as follow: Vendor-1 (36, 4, 12, 0.90, 0.75); Vendor-2 (36, 4, 107, 0.90, 0.25); Vendor-3 (21, 19, 52, 0.52, 0.28).

**Table 3:** Stratified summary statistics of three AI algorithms for detection of solid (3A, n = 127), ground glass nodules (3B, n = 76), and part-solid (3C, n = 40) lung nodules.

	Vendor 1	Vendor 2	Vendor 3
**3A: Solid lung nodules (n = 127)**			
True positives	105	96	53
False positives	16	184	82
False negatives	22	31	74
True negatives	29	6	14
Sensitivity	0.83	0.76	0.42
Recall	0.82	0.75	0.41
Precision	0.86	0.34	0.39
**3B: Ground glass nodules (n = 76)**			
True positives	53	50	1
False positives	11	154	53
False negatives	23	26	75
True negatives	34	4	17
Sensitivity	0.90	0.90	0.53
Recall	0.69	0.65	0.01
Precision	0.82	0.24	0.01
**3C: Part-solid nodules (n = 40)**			
True positives	36	36	21
False positives	12	107	52
False negatives	4	4	19
Ture Negatives	53	8	31
Sensitivity	0.70	0.66	0.01
Recall	0.90	0.90	0.52
Precision	0.75	0.25	0.28

There were wide variations in the ROC AUCs of different vendor algorithms for classifying lung nodules based on their attenuation. The ROC AUCs (with 95% confidence intervals) for detection of solid, groundglass, and part-solid nodules are summarized in Table 4.

**Table 4:** Summary of areas under the curve with 95% confidence interval (AUC 95% CI) for detection of solid, part-solid and ground-glass nodules with the three AI algorithms assessed in our study.

	AUC (95% CI)		
	Vendor 1	Vendor 2	Vendor 3
Solid nodules	0.74 (0.54–0.83)	0.61 (0.54–0.67)	0.74 (0.68–0.80)
Part solid nodules	0.86 (0.78–0.94)	0.52 (0.41–0.62)	0.55 (0.44–0.66)
Groundglass nodules	0.73 (0.63–0.82)	0.66 (0.58–0.74)	0.86 (0.80–0.93)

## Discussion

We were successful in designing, building, and testing a validation pipeline on three vendor applications with the goal of normalizing the performance measurement of independently developed algorithms, using a common test dataset of local clinical imaging studies and an evaluation module. Our evaluation successfully tested our data-preprocessing and algorithm assessment pipeline of tools from perspective of an image cohort creation and de-identification tool (DICOM receive and de-identification features from clinical to research environment); report and image annotation tools for ground-truth labeling (with multi-reader ground-truthing capabilities); server partitioning to receive vendor “black box” algorithm, and to enable model testing on our internal clinical data from within our security firewall (process local DICOM data with external ML algorithms without data sharing or upload to external clouds or software); model validation and result visualization tool (receive and adjudicate model outputs within the validation pipeline); and a performance assessment tool for calculating algorithm recall and precision (analyze and compare model outputs with ground-truthing to draw inference on local performance of individual ML algorithms).

There are several publications on development and evaluation of validation pipelines reported for both imaging and non-imaging ML algorithms [11–13]. These pipelines tackle various steps of ML algorithm development, data quality control, and performance from statistical analyses points of view. Versus other pipelines, the strengths of our validation infrastructure include incorporation and complete evaluation of multiple commercial ML algorithms from imaging data selection and de-identification, ground-truth labeling, ML processing within the institutional firewall, and statistical analysis and inferencing on comparative performance across multiple algorithms.

Vendor algorithm performance varied greatly, justifying our premise that a standardized objective ML algorithm evaluation process is needed. One vendor algorithm (vendor 3) was not able to detect ground-glass nodules, and as a result, their general performance suffered. However, even when assessing only solid nodule detection, this same vendor’s performance did not improve greatly. Another possible explanation for poor performance could be due to overfitting of the vendor model to a limited or homogeneous dataset upon which they trained their ML algorithm. Moreover, there is an obvious trade-off between sensitivity and specificity that requires adjustment during each algorithm model design, typically based on specific clinical needs. Vendors were instructed to tune their algorithms to an optimal intermediate configuration that would allow potential users to “plug and play” their tools without any kind of sensitivity-specificity calibration, allowing for a fair baseline comparison among the tools.

The major implication of our study included creation of validation pipeline for seamless processing and integration of AI outputs from multi-vendor AI systems. With an increasing number of the United States’ Food and Drug Administration (FDA)-cleared AI algorithms [14], it is important to perform an institutional point-of-use validation of AI algorithms before their clinical use. Indeed, the available information suggests that an overwhelming majority of these cleared algorithms (93/130) lacked prospective and/or multisite validation and testing [15]. Several publications have raised concern over lack of generalizability and robustness data for both the FDA-cleared as well as research AI algorithms [16]. Our validation pipeline could help identify potential reasons for under-performance of AI algorithms (such as different scanners, scan parameters, patient demographics, as well as performance with and without distracting or confounding findings).

Our validation pipeline pertained to the detection and characterization tasks of AI; therefore, modifications will be necessary to assess AI systems for triage, quantification, segmentation, and image processing. Scalability of such pipelines can also aid in evaluation of real-world surveillance and on-ground clinical performance of AI algorithms. The FDA sets minimum “pass criteria” or performance thresholds for AI algorithm based on their target application or predicate devices (AUC >0.95 for triage applications under the QFM product code) [17]. We recommend that prior to clinical integration and use, institutions should set or use similar standards on their local datasets so that AI results are trustworthy and reproducible. Exclusion of purely calcified nodules when assessing performance of nodule detection AI is important since calcified nodules are common, easier target to detect by both radiologists and AI, and most often do not result in change in patient care. Although characterization of nodules into calcified and non-calcified nodules is important, for assessing detection performance of AI algorithm, inclusion of such calcified nodules can “falsely” raise the performance of AI algorithm and not provide the “true” performance for detecting clinically meaningful non-calcified nodules.

The most difficult part of the validation pipeline is case selection and ground truthing. The former requires review of both radiology report, a robust radiology report search engine and an unbiased radiologist to further confirm appropriateness of selected cohorts both with and without target and distracting findings. Balancing the number of cases required for validation across a multitude of scanners, scan parameters with and without distractors, and the ground truth radiologists could be challenging at non-academic or extremely busy academic sites. The AI vendors in our study were pliant and cooperated with us to ensure a standardized output. However, standardization of AI output across multiple vendors from multiple parts of the world is not easy and might require intervention from the FDA or other stakeholder entities.

Limitations of our study include a data cohort of imaging examinations from one institution, although multiple scanner vendors, model, and protocol types were included. Exclusion criteria consisted of studies with findings of consolidation, significant pleural effusion or emphysematous change, architectural distortion, and post-surgical findings. While these findings can be commonly seen and should not be confounding or limiting factor for automatically detecting lung nodules, due to limitations of currently available narrow AI systems capable of detecting only single imaging features or findings, or systems trained using publicly available data sets that do not commonly contain studies presenting these types of findings, we opted to exclude studies with these findings. Since our objective was to assess differential detection abilities between solid, part-solid and groundglass nodules, we did not include distracting or confounding findings. However, for AI algorithms that target triage and lesion characterization, it is prudent to have representation of true negative (without any findings), potentially false positive (with distracting findings such as pneumonia or atelectasis for lung nodules), and cases where target findings exist with other imaging findings. In addition, lung nodule detection performance for diffusely calcified nodules was not assessed on this analysis since these findings are usually suggestive of benign etiology (e.g., granuloma or hamartoma), although also seen with metastases [18]. We acknowledge the need for creating data sets with the presence and different degrees of concomitant pulmonary findings to reflect the expected heterogeneity of studies that are seen in clinical practice where these systems will ultimately be used. Moreover, we envision the need for the creation of non-chest dedicated CT data sets that include in the field of view at least partially the lung parenchyma such as neck CT, cervical and thoracic spine CT, and abdominal CT, as well as in “regular” studies performed on dual-energy and positron emission tomography scanners (i.e., without maps or use of tracer, respectively) in which lung nodules can be found incidentally. A limitation of our pipeline pertains to manual or visual comparison of AI outputs with the ground truth although this task could be automated by comparing the intersection or overlap of annotation boxes drawn during ground truth and by the AI algorithms. However, such automation should ultimately require visual inspection for subtle nodules where extent of overlap can vary substantially (such as for groundglass and part-solid nodules), in multiple nodules that are in close proximity to one another (adjoining nodules could be counted as single nodules), or for bilobed nodules (one nodule counted could be counted as two nodules). Finally, for estimating model performance, the United States FDA recommends the estimation of ROC AUCs, which requires the inclusion of control or true negative cases. Although we did not include true negative cases (that is, cases without any nodules), we estimated ROC AUCs for detection of different nodule types (solid, ground glass, and part solid).

In summary, evaluation was done on our own clinical images without any data leaving our institutional firewall and without requiring third-party algorithm disclosure. The machine learning model validation pipeline we describe was tested in the specific use case of detecting lung nodules in chest CT examinations, but the developed techniques are easily generalizable to other types of tools now starting to be available from commercial entities.

## Abbreviations

AI: artificial intelligence
CAR/DS: computer-assisted reporting and decision support
FN: false negatives
FP: false positives
ML: machine learning
PACS: Picture archiving and communication system
TP: true positives
XML: Extensible Markup Language
